# Adenocarcinoma of the Rete Testis With PI3K/AKT/mTOR Pathway Alteration and a Gene Expression Profile Resembling Papillary Renal Cell Carcinoma: A Case Report

**DOI:** 10.1155/criu/8508136

**Published:** 2026-08-25

**Authors:** Sachein Sharma, Ryan Jaehne, Cyrus Oster, Dong Ren, Shivani Kandukuri, Li Lei, Dimitrios Korentzelos, Matthew J. Oberley, Eugene Muchnik, Mark G. Evans

**Affiliations:** ^1^ Caris Life Sciences, Phoenix, Arizona, USA, carislifesciences.com; ^2^ Pathology and Laboratory Medicine, UC Irvine School of Medicine, Irvine, California, USA, uci.edu; ^3^ Pathology, UC San Diego School of Medicine, La Jolla, California, USA, ucsd.edu; ^4^ Pathology, UPMC Shadyside, Pittsburgh, Pennsylvania, USA; ^5^ Hematology/Oncology, UPMC Susquehanna, Williamsport, Pennsylvania, USA

**Keywords:** adenocarcinoma of the rete testis, case report, genitourinary tumors, next-generation sequencing, papillary renal cell carcinoma, PI3K/AKT/mTOR pathway

## Abstract

Adenocarcinoma of the rete testis (ACRT) is a rare, gland‐forming neoplasm arising from rete testis epithelium and is typically associated with poor prognosis. Currently, our knowledge of ACRT has been limited to single reports and small series, and its molecular characteristics are largely unknown. We present a patient, whose scrotal tumor demonstrated a gene expression profile similar to that of papillary renal cell carcinoma (PRCC) and alteration of PI3K/AKT/mTOR pathway, thereby expanding our understanding of the genomic landscape of ACRT.

## 1. Introduction

Adenocarcinoma of the rete testis (ACRT) is a malignant tumor originating from rete testis epithelium. It is rare and there have been less than 100 described cases. Although this tumor has been reported in a wide age range, ACRT has an adult predominance and is most common in elderly White men, with a mean age of 54 years [1]. It has a nonspecific clinical presentation associated with signs of a mass lesion and symptoms of localized genital pain [2, 3]; the tumor may also be found in the context of hydrocele or incidentally in the setting of trauma [2–5]. ACRT has a poor prognosis and a 5‐year survival rate of approximately 15% [2, 6, 7]. Favorable prognostic factors include a tumor size less than 5 cm and tumor localized to the testis [2, 3].

ACRT originates from the testicular hilum and may directly extend to the testicular parenchyma, spermatic cord, and overlying skin [1, 2]. Histologically, it is identified by a visual demarcation between normal and neoplastic rete epithelium and is characterized by a spectrum of proliferative growth, including complex tubulopapillary, glandular, solid, cribriform, glomeruloid, retiform, sertoliform, kaposiform, micropapillary, and nested [1, 2]. By immunohistochemistry (IHC), tumor cells often express keratins, CD10, PAX8, calretinin, and WT1 [1, 2, 4, 8–10]. The genomic alterations responsible for ACRT are relatively unknown, with few studies featuring molecular analyses having been published [11, 12]. Here, we present an additional patient whose tumor underwent next‐generation sequencing, which revealed genomic changes in the PI3K/AKT/mTOR pathway.

## 2. Case Presentation

A 74‐year‐old White man presented with a chief complaint of abdominal pain. He had a history of prostate cancer managed with cryosurgery 7 years prior. His prostate‐specific antigen (PSA) level had been increasing over the previous 4 years, up to 0.87 ng/mL. Two months before his abdominal pain, a prostate‐specific membrane antigen (PSMA)‐PET scan was negative, but an MRI of the patient′s prostate showed a 3‐cm Prostate Imaging Reporting and Data System‐5 (PIRADS‐5) lesion in the bilateral anterior transition zone, bilateral anterior stroma and right anterior peripheral zone, and a 1.0‐cm PIRADS‐4 lesion in the left mid and medial peripheral zone. A transrectal ultrasound (TRUS) with multiple prostatic biopsies 1 month prior to presentation identified benign tissue. At the time of the abdominal pain, a CT of his abdomen and pelvis detected para‐aortic and aortocaval lymphadenopathy, but no discrete masses.

**Figure 1:**
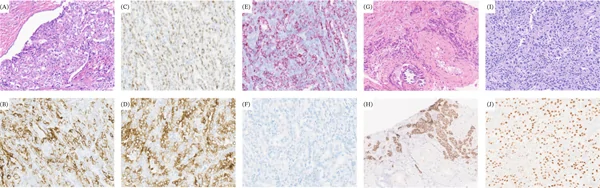
The patient′s testicular masses infiltrated the rete testis with (A) a prominent papillary growth pattern (hematoxylin and eosin staining, 200x magnification). By IHC (200x magnification), the cells expressed (B) CK7, (C) PAX8, (D) RCC, and (E) AMACR, and were negative for (F) NXK3.1. Some seminiferous tubules featured (G) clusters of cells with pleomorphic nuclei with condensed nuclear chromatin, inconspicuous nucleoli, and scant cytoplasm, with expression of (H) synaptophysin, suggestive of focal neuroendocrine differentiation. The patient′s subsequently excised CNS metastasis (hematoxylin and eosin staining, 200x magnification) featured (I) atypical cells with pleomorphic nuclei, moderately condensed chromatin, conspicuous nucleoli, and moderate amphophilic cytoplasm that expressed (J) PAX8 (200x magnification), consistent with the known ACRT.

The patient had also experienced 1 month of right scrotal swelling. An ultrasound of the scrotum identified multiple right testicular masses, and right orchiectomy was performed 1 month later. The morphology of the rete testis demonstrated benign epithelium transitioning to atypical, malignant glands (Figure 1A) displaying a predominantly papillary pattern. By IHC, the neoplastic cells expressed pancytokeratin, CK7, PAX8, RCC, and AMACR (Figure 1B–F) and were negative for CD117, CD30, OCT3/4, SALL4, glypican‐3, AFP, synaptophysin, chromogranin, WT1, calretinin, ER, AR, PSA, and NKX3.1, which excluded a diagnosis of metastatic prostatic adenocarcinoma and mesothelioma, and argued against a germ cell tumor. Fluorescence in situ hybridization (FISH) did not reveal increased Chr. 12p signal, as would be observed in carcinomas arising from germ cell tumors that harbor i12p. Focal cells (Figure 1G,H) adjacent to a dominant mass and localized to seminiferous tubules were found to express CD56, chromogranin, and synaptophysin, which resembled focal neuroendocrine differentiation with a mitotic rate of 1 to focally up to 10 per 10 high‐power fields. Collectively, these findings were most consistent with a diagnosis of ACRT, although the possibility of somatic transformation of a teratoma could not be entirely ruled out. FDG‐PET imaging completed 2 months after the right scrotal swelling first began identified metastases throughout his chest, abdomen, and pelvis. Cytopathology of a left cervical lymph node fine‐needle aspiration suggested metastatic ACRT, given that IHC studies of the neoplastic cells confirmed expression of CK7, CK20, and PAX8, as well as negativity for NKX3.1.

**Figure 2:**
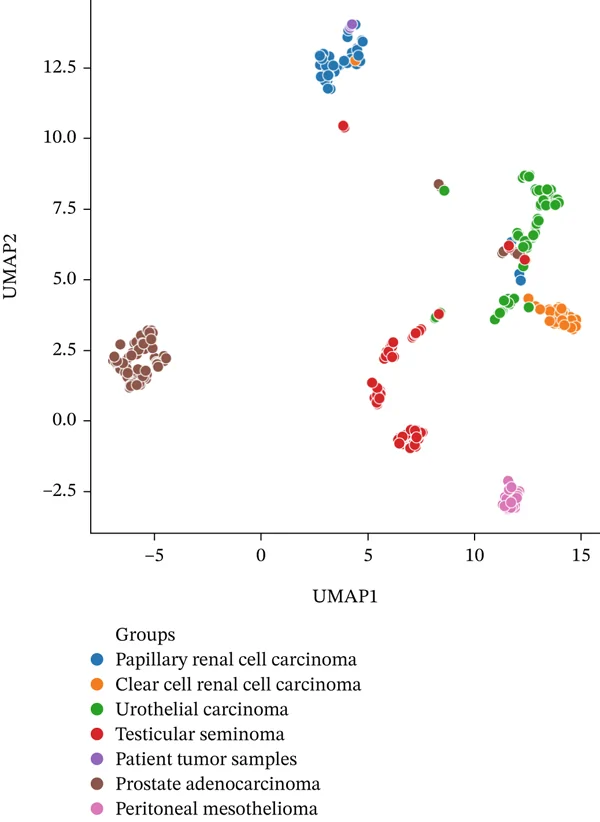
By gene expression UMAP clustering analysis, the patient′s tumor specimens clustered with cases of papillary renal cell carcinoma, separate from clear cell renal cell carcinoma, testicular seminoma, urothelial carcinoma, peritoneal mesothelioma, and prostatic adenocarcinoma.

The orchiectomy and lymph node specimens were submitted for whole exome and whole transcriptome sequencing at Caris Life Sciences. Testing of both tissue specimens identified the same *MTOR* p.K1981E likely pathogenic variant and two different *B2M* likely pathogenic variants (c.‐2_27del29 from the orchiectomy specimen and p.C45fs from the lymph node specimen). MI GPSai, a Genomic Prevalence Score developed by Caris Life Sciences that utilizes machine learning with genomic and transcriptomic data to elucidate tumor origin [13], suggested a papillary renal cell origin. Of note, no kidney mass has ever been identified. A Uniform Manifold Approximation and Projection (UMAP) was performed on RNA gene expression values obtained from whole‐transcriptome sequencing, specifically comparing the patient′s tumor specimens with cohorts of typical (formerly Type 1) papillary renal cell carcinoma (PRCC), clear cell renal cell carcinoma, testicular seminoma, bladder urothelial carcinoma, peritoneal mesothelioma, and prostatic adenocarcinoma that had been previously tested at Caris Life Sciences. This analysis revealed that the expression of the patient′s cancer closely associated with cases of PRCC (Figure 2). Of note, the UMAP reflects overall gene‐expression similarity and should not be interpreted as direct evidence of shared histogenesis.

Three months after presentation, he started palliative cisplatin and etoposide, as well as palliative radiation for retroperitoneal lymphadenopathy, but then developed a new mass in the central nervous system (CNS), which was surgically excised and was consistent with metastatic adenocarcinoma. IHC studies demonstrated that the malignant cells expressed MOC‐31, pancytokeratin, patchy CK7, CK20, and PAX8 (Figure 1H,J) and were negative for NKX3.1 and synaptophysin. The patient experienced a prolonged rehabilitation stay and completed adjuvant radiation to the metastatic tumor bed. Repeat FDG‐PET showed recurrent CNS disease and progressive lymphadenopathy in the mediastinal and supraclavicular nodes. Having been dismayed by the diagnosis and disheartened during the treatment, the patient succumbed to his disease before systemic treatment could be reinitiated.

## 3. Discussion

This case expands our understanding of the genomic landscape of ACRT. Molecular testing detected one *MTOR* likely pathogenic variant and two *B2M* likely pathogenic variants. The *MTOR* gene encodes a protein which belongs to the PI3K/AKT/mTOR pathway and helps to regulate cell metabolism, growth, and survival [14]. The protein encoded by the *B2M* gene is part of the major histocompatibility complex class I heavy chain that assists with peptide antigen presentation to the immune system [15]. These results add to those genomic alterations previously reported [11, 12] in ACRT, including pathogenic variants in *CDKN2A*, *AKT1*, *RB1*, *NF2*, *SETD2*, *TP53*, as well as amplification of *MET*. Although treatment is not yet biomarker‐associated, our identification of a second variant in the PI3K/AKT/mTOR pathway related to ACRT is hypothesis‐generating, as this suggests a role for targeted therapy; for example, direct AKT inhibition [16], mTOR inhibition [17, 18], and PI3K inhibition or PI3K and mTOR inhibition [19]. Currently, management is not standardized and includes surgery with adjuvant chemotherapy and/or radiotherapy.

Clarifying the presence of ACRT in our patient involved eliminating the possibility of alternative tumor types. The initial suspected diagnosis was involvement by the patient′s prior prostatic adenocarcinoma, given PSMA‐PET findings and a rising PSA level. Moreover, focal neuroendocrine differentiation in the patient′s orchiectomy specimen was observed, which could imply neuroendocrine transdifferentiation that has sometimes been described in prostate cancer following androgen‐deprivation therapy [20]. Our patient had not received this treatment, and IHC studies revealing no expression of NKX3.1, AR, and PSA were not consistent with metastasis of prostatic adenocarcinoma. Moreover, the focal neuroendocrine differentiation could have indicated somatic transformation of a teratoma or germ cell neoplasia in situ (GCNIS), but lack of staining for SALL4 and OCT3/4, as well as no evidence of i12p by FISH, argued against this disease process. Additionally, a diagnosis of serous cystadenocarcinoma was considered; although this neoplasm demonstrates morphologic differences, including serous‐type epithelium with psammoma body formations and an immunophenotype that includes ER and WT expression, which were not appreciated in our patient′s tumor. Similarly, other carcinomas such as mucinous, endometrioid, clear‐cell, and Brenner were also ruled out on morphologic and IHC grounds. Ultimately, molecular testing suggested an overlapping gene expression profile between our patient′s tumor and PRCC.

Although there are no disease‐defining biomarkers for ACRT, there are pertinent positive IHC findings (i.e., CK7, pancytokeratin, EMA, vimentin, CD10, BerEP4, CK5/6, MOC31, and PAX8) [1, 2, 4, 8–10] and pertinent negative IHC results (i.e., CD30, inhibin, NKX3.1, OCT3/4, PSA, PSAP, SALL4, and CK20) [1, 2, 21, 22]. Similarly, there are no lineage‐associated biomarkers of PRCC, but a prototypical trisomy/polysomy of chromosome 7, loss of chromosome Y, pathogenic variants in *MET* and *CDKN2A* have been described [23]. Likewise, there are pertinent positive IHC findings (i.e., AMACR, CK7, PAX8, CK AE1/AE3, EMA, CAM5.2, and vimentin) [24, 25] and pertinent negative IHC results (i.e., CAIX, CD117, WT1, CD57, and GATA3) [26, 27].

Considering the shared pertinent IHC findings (e.g., CK7+, pancytokeratin+, EMA+, vimentin+, PAX8+, RCC+, and AMACR+), it is not surprising that the respective primary sites of ACRT and PRCC have a common embryologic origin in the mesonephros that eventually forms genitourinary (GU) structures [28]. This observation may be explained by overlapping nephric transcription‐factor activity manifesting similar tubular epithelial phenotypes and activated oncogenic pathways [10, 12, 23, 29, 30]. Interestingly, in 1945, Feek and Hunter described one of the first cases of ACRT as “papillary carcinoma arising from rete testis” [31]. Therefore, in the GU tract, diagnosing ACRT as metastatic PRCC could be a potential pitfall, so clinical correlation is recommended and additional case reports or series are needed. Moreover, to our knowledge, comprehensive molecular profiling of ACRT has not yet been completed given the paucity of reported cases. IHC studies can assist in its identification, but ultimately further research is warranted into this rare tumor type.

## Funding

No funding was received for this manuscript.

## Disclosure

All authors have read and approved the final version of the manuscript. M.G.E., MD had full access to all of the data in this study and takes complete responsibility for the integrity of the data and the accuracy of the data analysis.

## Conflicts of Interest

S.S., R.J., C.O., M.J.O., and M.G.E. are full‐time employees of Caris Life Sciences.

## Supporting information


**Supporting Information** Additional supporting information can be found online in the Supporting Information section. 

## Data Availability

The data that support the findings of this study are available from the corresponding author upon reasonable request.
