# Supplementary material for: Adenocarcinoma of the Rete Testis With PI3K/AKT/mTOR Pathway Alteration and a Gene Expression Profile Resembling Papillary Renal Cell Carcinoma: A Case Report
Source: Case Rep Urol. 2026 Aug 25;2026:8508136. doi: 10.1155/criu/8508136 (PMC13504671; doi:10.1155/criu/8508136)
Supplement: Supplementary file 1 [file CRIU-2026-8508136-s001.pdf]

CARE case report guidelines

CARE Checklist of information to include when writing a case report

(cc) BY-NC-ND

Topic	Item	Checklist item description	Reported on Line
Title	1	The diagnosis or intervention of primary focus followed by the words "case report"	4 to 5
Key Words	2	2 to 5 key words that identify diagnoses or interventions in this case report, including "case report"	49 to 50
Abstract (no references)	3a	Introduction: What is unique about this case and what does it add to the scientific literature?	56 to 59
	3b	Main symptoms and/or important clinical findings	56 to 59
	3c	The main diagnoses, therapeutic interventions, and outcomes	56 to 59
	3d	Conclusion—What is the main "take-away" lesson(s) from this case?	56 to 59
Introduction	4	One or two paragraphs summarizing why this case is unique (**may include references**)	63 to 80
Patient Information	5a	De-identified patient specific information.	84
	5b	Primary concerns and symptoms of the patient.	84
	5c	Medical, family, and psycho-social history including relevant genetic information	84 to 85
	5d	Relevant past interventions with outcomes	85 to 93
Clinical Findings	6	Describe significant physical examination (PE) and important clinical findings.	95
Timeline	7	Historical and current information from this episode of care organized as a timeline	95 to 96
Diagnostic Assessment	8a	Diagnostic testing (such as PE, laboratory testing, imaging, surveys).	96 to 126
	8b	Diagnostic challenges (such as access to testing, financial, or cultural)	96 to 126
	8c	Diagnosis (including other diagnoses considered)	96 to 126
	8d	Prognosis (such as staging in oncology) where applicable	96 to 126
Therapeutic Intervention	9a	Types of therapeutic intervention (such as pharmacologic, surgical, preventive, self-care)	128 to 136
	9b	Administration of therapeutic intervention (such as dosage, strength, duration)	128 to 136
	9c	Changes in therapeutic intervention (with rationale)	128 to 136
Follow-up and Outcomes	10a	Clinician and patient-assessed outcomes (if available)	128 to 136
	10b	Important follow-up diagnostic and other test results	128 to 136
	10c	Intervention adherence and tolerability (How was this assessed?)	128 to 136
	10d	Adverse and unanticipated events	128 to 136
Discussion	11a	A scientific discussion of the strengths AND limitations associated with this case report	158 to 207
	11b	Discussion of the relevant medical literature **with references**.	158 to 207
	11c	The scientific rationale for any conclusions (including assessment of possible causes)	158 to 207
	11d	The primary "take-away" lessons of this case report (without references) in a one paragraph conclusion	203 to 207
Patient Perspective	12	The patient should share their perspective in one to two paragraphs on the treatment(s) they received	135 to 136
Informed Consent	13	Did the patient give informed consent? Please provide if requested	Yes ☑ No ☐
